# Rare genetic variants involved in multisystem inflammatory syndrome in children: a multicenter Brazilian cohort study

**DOI:** 10.3389/fcimb.2023.1182257

**Published:** 2023-07-31

**Authors:** Bárbara Carvalho Santos Dos Reis, Roberta Soares Faccion, Flavia Amendola Anisio de Carvalho, Daniella Campelo Batalha Cox Moore, Maria Celia Chaves Zuma, Desirée Rodrigues Plaça, Igor Salerno Filgueiras, Dennyson Leandro Mathias Fonseca, Otavio Cabral-Marques, Adriana Cesar Bonomo, Wilson Savino, Flávia Cristina de Paula Freitas, Helisson Faoro, Fabio Passetti, Jaqueline Rodrigues Robaina, Felipe Rezende Caino de Oliveira, Ana Paula Novaes Bellinat, Raquel de Seixas Zeitel, Margarida dos Santos Salú, Mariana Barros Genuíno de Oliveira, Gustavo Rodrigues-Santos, Arnaldo Prata-Barbosa, Zilton Farias Meira de Vasconcelos

**Affiliations:** ^1^ Programa de Pós Graduação em Pesquisa Aplicada à Saúde da Criança e da Mulher, Instituto Nacional de Saúde da Mulher, da Criança e do Adolescente Fernandes Figueira (IFF), Fundação Oswaldo Cruz (FIOCRUZ), Rio de Janeiro, RJ, Brazil; ^2^ Laboratório de Alta Complexidade (LACIFF), Unidade de Pesquisa Clínica, IFF, FIOCRUZ, Rio de Janeiro, RJ, Brazil; ^3^ Departamento de Imunologia, IFF, FIOCRUZ, Rio de Janeiro, RJ, Brazil; ^4^ Unidade de Pacientes Graves, Departamento de Pediatria, IFF, FIOCRUZ, Rio de Janeiro, RJ, Brazil; ^5^ Faculdade de Medicina, Universidade Federal Fluminense, Niterói, Rio de Janeiro, RJ, Brazil; ^6^ Departamento de Análises Clínicas e Toxicológicas, Faculdade de Ciências Farmacêuticas (FCF), Universidade de São Paulo (USP), São Paulo, SP, Brazil; ^7^ Programa de Pós-Graduação em Farmácia (Fisiopatologia e Toxicologia), FCF, USP, São Paulo, SP, Brazil; ^8^ Departamento de Imunologia, Instituto de Ciências Biomédicas (ICB), USP, São Paulo, SP, Brazil; ^9^ Programa Interunidades de Pós-graduação em Bioinformática, Instituto de Matemática e Estatística (IME), USP, São Paulo, SP, Brazil; ^10^ Network of Immunity in Infection, Malignancy, and Autoimmunity (NIIMA), Universal Scientific Education and Research Network (USERN), São Paulo, Brazil; ^11^ Department of Medicine, Division of Molecular Medicine, University of São Paulo School of Medicine, São Paulo, SP, Brazil; ^12^ Laboratory of Medical Investigation 29, University of São Paulo School of Medicine, São Paulo, Brazil; ^13^ Laboratoírio de Pesquisas Sobre o Timo, Instituto Oswaldo Cruz (IOC), FIOCRUZ, Rio de Janeiro, RJ, Brazil; ^14^ Instituto National de Ciencia e Tecnologia em Neuroimunomodulação (INCT/NIM), IOC, FIOCRUZ, Rio de Janeiro, RJ, Brazil; ^15^ Rede FAPERJ de Pesquisa em Neuroinflamação, IOC, FIOCRUZ, Rio de Janeiro, RJ, Brazil; ^16^ Rede INOVA-IOC em Neuroimunomodulação, IOC, FIOCRUZ, Rio de Janeiro, RJ, Brazil; ^17^ Laboratório de Regulação da Expressão Gênica, Instituto Carlos Chagas (ICC), FIOCRUZ, Curitiba, PR, Brazil; ^18^ Instituto D’Or de Pesquisa e Ensino (IDOR), Rio de Janeiro, Brazil; ^19^ Pediatric Intensive Care Unit, Hospital Alvorada Moema, São Paulo, SP, Brazil; ^20^ Pediatric Intensive Care Unit, Hospital Martagão Gesteira, Salvador, BA, Brazil; ^21^ Pediatric Intensive Care Unit, Hospital Universitário Pedro Ernesto (HUPE), Universidade do Estado do Rio de Janeiro (UERJ), Rio de Janeiro, Brazil; ^22^ Pediatric Intensive Care Unit, Instituto de Puericultura e Pediatria Martagão Gesteira (IPPMG), Universidade Federal do Rio de Janeiro (UFRJ), Rio de Janeiro, Brazil

**Keywords:** multisystem inflammatory syndrome in children, pediatric inflammatory multisystem syndrome, coronavirus infection, mucocutaneous lymph node syndrome, Kawasaki disease, whole exome sequencing, whole exome sequencing

## Abstract

**Introduction:**

Despite the existing data on the Multisystem Inflammatory Syndrome in Children (MIS-C), the factors that determine these patients evolution remain elusive. Answers may lie, at least in part, in genetics. It is currently under investigation that MIS-C patients may have an underlying innate error of immunity (IEI), whether of monogenic, digenic, or even oligogenic origin.

**Methods:**

To further investigate this hypothesis, 30 patients with MIS-C were submitted to whole exome sequencing.

**Results:**

Analyses of genes associated with MIS-C, MIS-A, severe covid-19, and Kawasaki disease identified twenty-nine patients with rare potentially damaging variants (50 variants were identified in 38 different genes), including those previously described in IFNA21 and IFIH1 genes, new variants in genes previously described in MIS-C patients (KMT2D, CFB, and PRF1), and variants in genes newly associated to MIS-C such as APOL1, TNFRSF13B, and G6PD. In addition, gene ontology enrichment pointed to the involvement of thirteen major pathways, including complement system, hematopoiesis, immune system development, and type II interferon signaling, that were not yet reported in MIS-C.

**Discussion:**

These data strongly indicate that different gene families may favor MIS- C development. Larger cohort studies with healthy controls and other omics approaches, such as proteomics and RNAseq, will be precious to better understanding the disease dynamics.

## 1 Introduction

At the end of 2019, the first reports of a new respiratory infectious disease appeared, especially severe in older adults and adults with comorbidities, which developed into a pandemic and became known worldwide as COVID-19. Children appeared to be spared, developing an asymptomatic or oligosymptomatic clinical presentation. Rapidly it was identified that the disease was caused by a coronavirus (SARS-CoV-2). Soon after the onset of the pandemic, children with fever, gastrointestinal symptoms, Kawasaki-like manifestations, acute cardiac involvement, and a hyperinflammatory state started to be reported worldwide (Dufort et al., 2020; Feldstein et al., 2020; Riphagen et al., 2020; Verdoni et al., 2020; Lima-Setta et al., 2021). These manifestations were not caused directly by the SARS-CoV-2, as the peak of reported cases occurred 2-6 weeks after the peak of COVID-19 cases (Dufort et al., 2020; Feldstein et al., 2020; Belay et al., 2021; Lima-Setta et al., 2021). Early reports drew attention to the resemblance between the new disorder and other known conditions such as Kawasaki disease (KD), toxic shock syndrome (TSS), and hemophagocytic lymphohistiocytosis (HLH). The disease, now known as multisystem inflammatory syndrome in children (MIS-C), is characterized as a post-viral complication of unknown pathophysiology associated with *SARS-CoV-2* infection. The disease is rare and has also been described in newborns and adults, receiving different designations: MIS-N and MIS-A, respectively (Patel et al., 2021; Shaiba et al., 2022).

The clinical manifestations of COVID-19 vary enormously, from silent infection to lethal disease. Thus, genetic factors have been suspected to be involved in the severity of the disease, especially in young people. Studies on autoantibodies and gene *loci* with rare genetic variants related to type I interferons (IFNs) were reported in adult cases (Bastard and Rosen, 2020; Zhang and Bastard, 2020). Those were followed by the identification of single nucleotide polymorphisms (SNPs) that increase susceptibility to severe COVID-19 in genes such as *TLR7* (van der Made et al., 2020; Asano et al., 2021; Solanich et al., 2021).

Being a rare and heterogeneous disease, it is currently under investigation if genetic factors may also be determinants in the evolution of MIS-C. It is possible that lower penetrance or hypomorphic mutations represent an underlying factor, at least in part of these cases, triggering the phenotype of a pro-inflammatory insult, although not capable of doing so *per se* (Schulert and Cron, 2020).

Here we describe rare genetic variants identified in a multicenter Brazilian cohort of 30 MIS-C patients and signaling pathways associated with these genes. The data reported herein strongly indicate that different gene families may favor MIS-C development.

## 2 Materials and methods

### 2.1 Design, settings, and participants

This cross-sectional analysis was conducted with 30 pediatric patients from eight institutions – six private and two public – from three states in Brazil.

All subjects who met the following CDC case definition for MIS-C (00432, H.A, 2021) were included: 1) fever >38.0 °C for ≥24h (objective or subjective); 2) with multisystem (>2) organ involvement (cardiac, renal, respiratory, hematologic, gastrointestinal, dermatologic or neurological); 3) laboratory evidence of inflammation, including, but not limited to, one or more of the following: high values of C-reactive protein (CRP), erythrocyte sedimentation rate (ESR), fibrinogen, procalcitonin, d-dimer, ferritin, lactic acid dehydrogenase (LDH), or interleukin 6 (IL-6); elevated neutrophils, reduced lymphocytes, and low albumin; 4) no plausible alternative diagnosis; 5) current or recent SARS-CoV-2 infection diagnosed by a positive reverse transcription followed by quantitative polymerase chain reaction (RT-qPCR) or positive serological tests (IgM, IgG or IgA), or exposure to a suspected or confirmed COVID-19 case within the four weeks prior to the onset of symptoms.

The analyses included clinical and laboratory data collected from patients with disease onset from May 2020 to January 2022. Patients were coded chronologically according to their entrance into the study. All recruited subjects underwent whole exome sequencing, and there were no losses due to poor sample quality or refusal to participate.

### 2.2 Data collection

In cases where the patient had already been discharged from the hospital, the patient and his/her guardian were contacted and invited to participate in the research. When the patient was still hospitalized at the moment of recruitment, during hospitalization, the patient and his/her guardian were invited to participate in the research. In both cases, an informed consent form was presented and signed, followed by blood sample collection that was sent for DNA extraction and whole exome sequencing (WES). For each subject, a medical record review from the intensive care unit was also performed to fill out a specific RedCap electronic form (**Supplementary Table 1**).

### 2.3 Whole exome sequencing and analysis of genetic variants

#### 2.3.1 Genomic DNA preparation

Peripheral blood was collected from all patients in heparin tubes, and genomic DNA was extracted from patients *via* peripheral blood leukocytes using the PureLink^®^ Genomic DNA mini kit (Thermo Fisher Scientific) according to the manufacturer’s protocol. The DNA concentration in the samples was assessed by fluorimetry using the Invitrogen Qubit^®^ 4 fluorimeter (Thermo Fisher Scientific). Samples were stored in a biorepository at -80°C until submitted to WES.

#### 2.3.2 Library preparation and sequencing

The DNA libraries were prepared with 50 ng of DNA. Initially an enzymatic fragmentation of the material was performed in portions with an average of 150 bp, subsequently analyzed through the Bioanalyzer software (Agilent). DNA libraries were prepared using Illumina Exome Panel (45Mb) and sequenced on the NextSeq2000 platform to generate 100 bp paired-end reads (2 x 100 bp) at the sequencing facility Centro de Genomica Funcional (ESALQ/USP, Piracicaba, Brazil).

#### 2.3.3 Variant calling and analysis

Reads were processed using the nf-core pipeline sarek aligned to the human reference genome GRCh37 (Garcia et al., 2020). Variants were filtered using panels with genes related to different diseases built from literature data. Since the pathophysiology of MIS-C is still elusive, panels with genes related to severe COVID-19 (van der Made et al., 2020; Zhang and Bastard, 2020; Asano et al., 2021; Solanich et al., 2021), MIS-C (Lee et al., 2020; Chou et al., 2021; Abolhassani et al., 2022b; Abuhammour et al., 2022; Vagrecha et al., 2022), MIS-A (Ronit et al., 2021), and KD (Burgner et al., 2009; Tsai et al., 2011; Lee et al., 2012; Onouchi et al., 2012) were applied, in addition to genes related to inborn errors of immunity (IEI). For this, we used the gene list from the 2021 update of the International Union of Immunological Societies Expert Committee (IUIS) for Human Inborn Errors of Immunity (Bousfiha et al., 2020; Tangye et al., 2021). Gene panels are depicted in **Supplementary Table 2**.

During analysis of candidate variants (CV), variants with allelic frequency ≤ 5% were selected using the Exome Aggregation Consortium database (ExAC), 1000 Genomes Project database, gnomAD (aggregated), and ABraOM, a Brazilian online mutation archive (http://abraom.ib.usp.br/). To evaluate the pathogenicity of the variants, thirteen predictors were considered, according to the following bioinformatics tools: CADD, BayesDel_addAF, DANN, DEOGEN2, EIGEN, FATHMM-MKL, LIST-S2, M-CAP, MutationAssessor, MutationTaster, SIFT, Polyphen-2, and PrimateAI. Respective websites are seen in **Supplementary Table 3**. Rare variants classified as damaging/deleterious in at least one of the *in silico* predictors were selected. The clinical relevance of the variants was evaluated using ClinVar, Polymorphism database (dbSNP), and Human Gene Mutation Database (HGMD). The results are presented in the tables below.

#### 2.3.4 Functional enrichment, pathway, and protein-protein interaction analyses

To identify the functional relationships between genes with CV and the biological processes (BP) that they are involved in, gene ontology (GO) pathway enrichment and protein-protein interaction (PPI) network analysis were performed in String (Szklarczyk et al., 2021). First, network nodes were plotted with the identified genes. Then, a rough estimation of possibly disrupted signaling pathways was obtained by applying an interaction score of medium confidence (0.4). Following the unsupervised analysis, supervised clustering of relevant pathways was performed, and Circos graphs were generated in the circlize R package in R version 4.0.5 (Gu et al., 2014).

#### 2.3.5 Ethical aspects

The study was submitted to the Internal Review Board (IRB) of Instituto D’Or de Pesquisa e Ensino - IDOR (Proponent Institution) under CAAE n° 30272920.0.1001.5249 and was approved. Each participating center evaluated and approved the project, through its own IRB or the associated IRB.

## 3 Results

### 3.1 Demographic, clinical, and laboratory data

Thirty patients were included in the study. Ages ranged from 0 to 17 years. The median age of patients was 6 (Q1-Q3 = 3-8.75) years; 63.3% of the patients were female; 58.3% were white; and only 10.0% had comorbidities – asthma was the most common (6.6%) (**Table 1**).

**Table 1:** Demographic, clinical and laboratory data of the MIS-C patients.

Characteristics	N	%
Age (years), median, IQR	6	3-8.75
Race		
Caucasian	14	58.3
Brown	6	25.0
Black	4	16.6
Sex		
Male	11	36.6
Female	19	63.3
Comorbidities	3	10
Asthma	2	6.6
Malnutrition	1	3.3
Obesity	1	3.3
Onco-hematological disease	1	3.3
Phenotype		
Incomplete Kawasaki Disease	4	13.3
Toxic Shock Syndrome	1	3.3
Acute Cardiac Dysfunction	14	46.6
Kawasaki-like Disease	10	37.0
Epidemiological link with SARS-CoV-2	16	59.2
Rapid Serological Test		
Positive IgM	6/7	85.7
Positive IgG	5/7	71.4
RT-PCR for SARS-CoV-2		
SARS-CoV-2 detected	7/18	38.8
Serology (ELISA)		
Positive IgM	0/5	0
Positive IgA	17/19	89.4
Positive IgG	17/20	85.0
Clinical and Laboratory Findings		
Gastrointestinal symptoms	21	70.0
Abdominal pain	17	56.6
Diarrhea	9	30.0
Emesis	12	40.0
Respiratory symptoms	5	16.6
Kawasaki-like manifestations	21	70.0
Skin rash	18	60.0
Conjunctivitis	14	46.6
Mucositis	6	20.0
Lymphadenopathy	6	20.0
Signs of shock	24	80.0
Hypotension	14	46.6
Tachycardia	16	53.3
Slow capillary refill	11	36.6
Cutaneous pallor	7	23.3
Cold extremities	5	16.6
Low urine output	10	33.3
Metabolic acidosis	6	20.0
Increased lactate	4	13.3
Acute renal failure	7	23.3
Hepatic injury	6	20.0
Oxygen therapy needed	10	33.3
Invasive mechanical ventilation	8	26.6
Laboratory analysis		
Increased markers of cardiac dysfunctions	25*	92.5
Anemia	15	50.0
Leukocytosis (> 15 × 10^9^/L)	17	56.6
Lymphopenia (< 1.5 × 10^9^/L)	22	73.3
Thrombocytosis (> 450 × 10^9^/L)	6	20.0
Thrombocytopenia (< 150 × 10^9^/L)	17	56.6
Hypoalbuminemia (< 3 g/dL)	19	63.3
Other Clinical Findings	Median	IQR
Fever (days)	6	5.75-8
Invasive mechanical ventilation (days)	6	4.75-8

IQR, interquartile range. * Only 27 patients were tested.

The median duration of fever was six days (Q1-Q3 = 5.75-8), and the most frequent symptoms were skin rash (60.0%), followed by abdominal pain (56.6%), tachycardia (53.3%), conjunctivitis and hypotension (both present in 46.6%). Signs of shock were described in 80.0% of patients, gastrointestinal symptoms in 70.0%, and Kawasaki-like symptoms in almost 70.0%. Most patients had acute cardiac dysfunction (46.6%), followed by the Kawasaki-like phenotype (37.0%) (**Table 1**). None of the patients reported prior history that might suggest an IEI, autoimmune, or autoinflammatory disease.

A history of contact with a suspected case of COVID-19 was identified in 59.2% of cases. Most individuals with available serologic testing results had positive IgA and IgG titers for SARS-CoV-2 (89.4% and 85.0% of patients, respectively). In addition, a n RT-PCR-detectable SARS-CoV-2 viral load was identified in 38.8% of the patients (**Table 1**). The most common SARS-CoV-2 variant of concern (VOC) at the time of diagnosis of each case is depicted in **Supplementary Table 4**.

### 3.2 WES analysis

A total of 50 CVs in 38 genes were identified in 29 patients (96.6%). Thirty-three genes were comprised in the IEI panel, three in the KD panel, one in the COVID-19 panel, and seven were part of the MIS-C panel. Six genes (15.7%) identified were in more than one gene panel.

The most frequently altered gene was *HLA-A*. Nine of the 30 patients had multiple *HLA-A* variants, corroborating the hypothesis that MIS-C encompasses an autoimmune basis. The same *G6PD* and two different *PFR1* CV were detected in five children. The same *APOL1* variant, two different *CFB* variants, and the same *FCN3* CV were detected in three children. Two different *CFTR*, a single *JAK3*, a single *KMT2D*, a single *PMS2*, a single *TNFRSF13B*, and a single *TP53*, were detected in two patients. The other CV were detected in only one patient.

One patient harbored CV in six of the evaluated genes (P20), four patients in 4 genes (P11, P19, P21, and P26), and five patients in 3 genes (P1, P5, P12, P13, and P24). In the other patients, only one or two CVs were detected, except in P30, in whom no CV was detected. These results are summarized in **Table 2** and **Supplementary Table 5**.

**Table 2:** Genetic pathogenic and likely pathogenic variants identified in immune-related genes.

Gene	Patient	Transcript	Variant	Effect	Gene Panel	AF (GnomAD aggregated)	AF (ABraOM)	Inheritance Pattern (OMIM*)	AF (Internal cohort^#^)	dbSNP ID
APOL1	P7, P16, P20	NM_003661.4	c.1164_1169delTTATAA	Frameshift	IEI	1.31%	2.87%	UN	1.83%	rs71785313
BRCA2	P3	NM_000059.3	c.1138del	Frameshift	IEI	N/A	N/A	AR/AD/UN	0.31%	rs80359264
C6	P13	NM_000065.3	c.1879del	Frameshift	IEI	0.11%	0.29%	AR	0.31%	rs61469168
C8A	P24	NM_000562.2	c.1413_1416del	Frameshift	IEI	N/A	N/A	AR	0.31%	N/A
CD46	P19	NM_002389.4	c.857-2A>C	Splice acceptor	IEI	< 0.01%	N/A	AR/AD	0.31%	rs773618613
CFB	P5, P11	NM_001710.5	c.26T>A	Missense	IEI	3.83%	3.76%	AR/AD/UN	1.22%	rs4151667
CFB	P12	NM_001710.5	c.94_95delinsTA	Stop gained	IEI	3.83%	N/A	AR/AD/UN	1.22%	rs4151667
CFHR1	P26	NM_002113.2	c.790 + 1G>A	Splice donor	IEI	0.25%	0.46%	AR/AD	0.31%	rs140799744
CFHR3	P21	NM_021023.5	c.796 + 1G>A	Splice donor	IEI	0.2%*	0.09%	AR/AD	0.61%	rs370108606
CFHR5	P20	NM_030787.3	c.1704T>A	Stop gain	IEI	0.26%	0.34%	AD	0.31%	rs143140599
CFTR	P12	NM_000492.3	c.1521_1523del	Deletion	IEI	0.72%	0.43%	AR/AD	0.31%	rs113993960
CFTR	P13	NM_000492.3	c.617T>G	Missense	IEI	0.72%	0.17%	AR/AD	0.31%	rs113993960
CLPB	P1	NM_001258392.2	c.1132A>G	Missense	IEI	0.02%	N/A	AR/AD	0.31%	rs144078282
CTC1	P5	NM_025099.5	c.248_251dup	Frameshift	IEI	< 0.01%	N/A	AR	0.31%	rs745467709
DCLRE1C	P24	NM_001033855.2	c.959C>G	Missense	IEI	0.36%	0.77%	AR	0.31%	rs41298896
ERAP1	P24	NM_016442.3	c.170_173delACGT	Frameshift	Kawasaki	0.03%	N/A	N/A	0.31%	rs546509120
ERCC6L2	P19	NM_020207.7	c.4095delA	Frameshift	IEI	N/A	N/A	AR	0.31%	N/A
FANCA	P9	NM_000135.2	c.3349-1G>C	Splice acceptor	IEI	N/A	N/A	AR	1.82%	rs769862233
FCN3	P6, P16, P18	NM_003665.3	c.349del	Frameshift	IEI	1.62%	3.03%	AR	0.92%	rs532781899
G6PD	P5, P21, P23, P26, P28	NM_001360016.2	c.202G>A	Missense	Kawasaki	1.16%	4.02%	XL/UN	1.83%	rs1050828
HAVCR2	P28	NM_032782.4	c.291A>G	Missense	Kawasaki	0.29%	0.34%	AR	0.31%	rs35960726
HLA-A	P1, P8, P9, P14, P26	NM_002116.7	c.265_266del	Frameshift	Kawasaki	0.33%	N/A	UN	0.92%	rs1491212130
HLA-A	P1, P8, P9, P14, P26	NM_002116.7	c.268_269insG	Frameshift	Kawasaki	0.01%	N/A	UN	0.92%	rs576213756
HLA-A	P1, P8, P14, P26	NM_002116.7	c.272_273insC	Frameshift	Kawasaki	0.01%	1.24%	UN	0.92%	rs775079480
HLA-A	P1, P8, P9, P14, P26	NM_002116.7	c.295_296insTA	Frameshift	Kawasaki	0.01%	N/A	UN	0.92%	rs774211961
HLA-A	P1, P8, P14, P26	NM_002116.7	c.301_302del	Frameshift	Kawasaki	0.01%	N/A	UN	0.92%	rs750632693
HLA-A	P8	NM_002116.7	c.626_627dup	Frameshift	Kawasaki	0.26%	N/A	UN	1.82%	rs199474609
HLA-A	P19, P20	NM_002116.8	c.619G>T	Missense	Kawasaki	0.05%	N/A	UN	0.61%	rs62687162
HLA-A	P2, P25	NM_002116.7	c.257delA	Frameshift	Kawasaki	0.01%	N/A	UN	0.61%	rs200082950
HLA-A	P2, P25	NM_002116.7	c.261delG	Frameshift	Kawasaki	0.01%	N/A	UN	0.61%	rs199474423
HLA-A	P2, P25	NM_002116.7	c.265_266insACAGATCT	Frameshift	Kawasaki	0.01%	N/A	UN	0.61%	rs752540700
IFIH1	P4	NM_022168.3	c.1641 + 1G>C	Splice donor	IEI	0.67%	1.28%	AR/AD/UN	0.31%	rs35337543
IFNA21	P29	NM_002175.2	c.376G>T	Stop gain	MIS-C	0.05%	0.13%	N/A	0.31%	rs146777169
IRF3	P10	NM_001197122.1	c.1231C>T	Stop gain	IEI/Covid-19	0.03%	N/A	AD	0.31%	rs149842990
JAK3	P1, P17	NM_000215.3	c.2164G>A	Missense	IEI	0.84%	0.94%	AR	0.61%	rs3213409
KMT2D	P22	NM_003482.3	c.2533del	Frameshift	IEI	N/A	N/A	AD	0.31%	rs767415197
MASP2	P4, P17	NM_006610.3	c.359A>G	Missense	IEI	2.18%	2.05%	AR	0.61%	rs72550870
PMS2	P11, P27	NM_000535.6	c.2186_2187del	Frameshift	IEI	<0.01%	1.33%	AR/AD	0.61%	rs587779335
POLE	P11	NM_006231.3	c.2864 + 1G>C	Splice donor	IEI	N/A	N/A	AR/AD	0.31%	N/A
PRF1	P11, P19	NM_001083116.1	c.272C>T	Missense	IEI	2.92%	4.96%	AR/UN	0.61%	rs35947132
PRF1	P13, P20	NM_001083116.1	c.11G>A	Missense	IEI	0.84%	1.92%	AR/UN	0.92%	rs35418374
RAD51	P21	NM_002875.4	c.449G>A	Missense	IEI	0.05%	0.17%	AD	0.61%	rs121917739
RANBP2	P27	NM_006267.4	c.7309_7311delinsTAA	Missense	IEI	N/A	N/A	AD	0.31%	N/A
SH3BP2	P26	NM_001145855.1	c.221G>A c.137G>A	Stop gain	IEI	N/A	N/A	AD	0.31%	N/A
SPINK5	P2	NM_006846.3	c.2468dup	Frameshift	IEI	0.13%	N/A	AR	0.31%	rs565782662
TERC	P7	NR_001566.1	n.58G>A	Regulatory	IEI	0.52%	1.54%	AD	1.82%	rs113487931
TERT	P20	NM_198253.2	c.1234C>T	Missense	IEI	0.32%	0.17%	AR/AD/UN	0.31%	rs34094720
TNFRSF13B	P15, P29	NM_012452.2	c.204dup	Frameshift	IEI	0.04%	N/A	AR/AD/UN	0.61%	rs72553875
TP53	P21, P22	NM_000546.5	c.467G>A	Frameshift	IEI	<0.01%	N/A	AR/AD/UN	0.92%	rs371524413
ZFHX3	P20	NM_006885.3	c.10536_10537del	Frameshift	Kawasaki	N/A	N/A	UN	0.31%	N/A

AF, allele frequency; IEI, inborn errors of immunity; UN, NA, not available; AR, recessive; AD, autosomal dominant; XL, x-linked. *According to OMIM (Online Mendelian Inheritance in Man). # Internal allele frequency from our research group cohort of immunological disease patients with 303 patients in total.

Regarding possible haplotypes, P11 and P12 both had variants in *CFB* and *PRF1*. P12 and P13 both had variants in *CTRF* and *PRF1*. All five patients with *PFR1* variants also harbored variants in at least one complement system gene associated with an IEI. We were unable to identify any association between CV profile and most frequent VOC at the time of MISC diagnosis and between patient clinical phenotype (**Supplementary Tables 4**, **5**).

### 3.3 Gene ontology and biological process analyses

**Figure 1:**
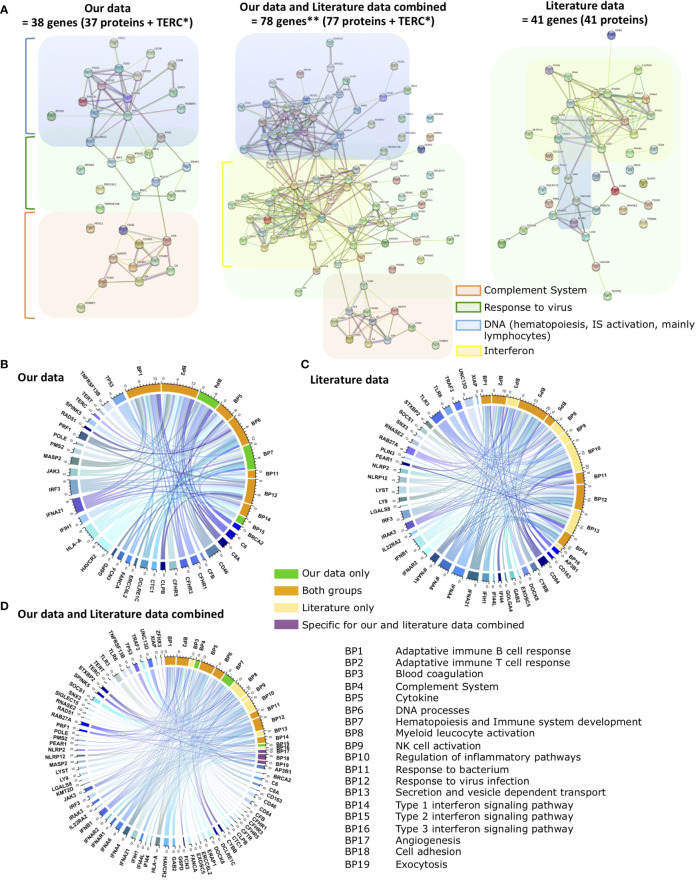
Functional relationships between genes with CV (candidate variants) in MISC patients. **(A)** Protein-Protein Interaction (PPI) Networks based on experimental evidence and expert-curated databases. Left panel: genes with CV identified in our study. Middle panel: genes with CV identified in our study plus genes with CV previously reported in literature. Right panel: genes with CV previously reported in literature. Genes that encode Complement System proteins are in orange, proteins in Response to virus pathways in green, including interferon pathway genes (yellow), and genes that regulate DNA processes in blue. IS: Immune system. Gene network analyses performed in String. Panels **(B–D)** depict Chord diagrams illustrating the functional relationships between the genes of **(B)** our study, **(C)** literature, and **(D)** our data and literature data combined and the biological processes (BP) that enrich them according to GO functional enrichment analysis. BPs are indicated by numbers. BPs enriched only by our study genes are in green, BPs enriched only by the literature genes in yellow, BPs that are enriched for both gene sets in orange, and BPs that only appear enriched with the combination of our genes with literature genes in purple. The size of the rectangles is proportional to the involvement of genes in the multiple pathways. Genes from each data set that does not enrich any BP does not appear in the images. The scale outside the circles indicates the number of relationships for each rectangle. Images created using circlize R package in R version 4.0.5. Colors for pathways, genes, and BPs are arbitrary. *Because TERC is not translated into a protein, String Nodes analysis does not include this gene. **We identified 38 genes with CV in MISC patients and other groups had previously described 41 genes. Because we identified CV in 3 genes that had already been described, accounting for those, the current total of genes with CV regarding MISC is 78 genes.

To identify relationships between genes that could indicate possible signaling pathways disruptions in MIS-C, we performed a gene network analysis of CV-bearing genes in String (Szklarczyk et al., 2021). Protein-Protein Interaction (PPI) Networks based on experimental evidence and expert-curated databases are displayed in **Figure 1A**. As observed in the left panel, our analyses identified CV in 38 genes, with 37 nodes representing proteins and 73 edges representing confidence level (expected number of edges = 19), with a PPI enrichment *p*-value <1.0^−16^. These genes can be roughly split into three functional categories as genes that encode complement system proteins (red), genes that encode proteins in “response to virus” pathways (green), including genes related to the interferon pathway (yellow), and genes that regulate DNA processes (blue). Importantly, these DNA processes are mostly linked to hematopoiesis, and immune system activation, mainly lymphocytes.

Other research groups previously identified 41 genes with CV in MIS-C patients (right panel) (Lee et al., 2020; Chou et al., 2021; Ronit et al., 2021; Abolhassani et al., 2022b; Abuhammour et al., 2022; Vagrecha et al., 2022). PPI Network analysis of these genes yields 41 nodes representing proteins and 93 edges representing confidence level (expected number of edges = 9), with a PPI enrichment *p*-value <1.0^−16^. Following the PPI Network analysis, it is observed that these literature-reported genes are largely related to response to virus pathways, several are interferon pathway- related genes, and a few are linked to DNA processes. When our data and literature data are combined (middle panel), there are 78 genes, 77 nodes representing proteins, and 192 edges representing confidence level (expected number of edges = 50), with a PPI enrichment *p*-value <1.0^−16^. All categories remained evident, complement system, DNA processes, and interferon gravitating around the main response to the virus response group. This is coherent with the importance of these genes in ensuring an appropriate immune response to COVID-19 infection, consistent with impairment of adequate infection resolution in MIS-C patients, and depicts a complementarity between genes described in previous studies and genes firstly associated with MIS-C in the present study.

To estimate more precisely which signaling pathways the CV we identified converge to impact on, hence would be more disrupted by pathological mutations in the described genes, we performed a Biological Processes (BP) Enrichment in String. The non-supervised analysis identified 86 BP with statistical significance (Confidence Interval of 95%), 35 of which can be further subcategorized into ten different functional groups (**Figure 1B**, **Table 3**, and **Supplementary Table 6**). To compare with previous findings, we performed the same analysis with the 41 previously described genes and identified 101 BP with statistical significance, 48 of which can be further subcategorized into 13 different functional groups (**Figure 1C**, **Table 3**, and **Supplementary Table 6**).

**Table 3:** String Enrichment non-supervised analysis of Biological Processes involving the identified genes, followed by supervised further categorization displaying genes in each group of pathways.

Pathways	Genes in non-supervised Biological Processes		
	Identified on the present study	Previously described by other groups	Identified on the present study and previously described
Pathways identified exclusively by genes described on the present study			
*Complement system*	C6, C8A, CD46, CFB, CFHR1, CFHR5, FCN3, MASP2	–	C6, C8A, CD46, CFB, CFHR1, CFHR3, CFHR5, FCN3, MASP2
*Hematopoiesis and immune system development*	BRCA2, CTC1, DCLRE1C, G6PD, HAVCR2, IFNA21, JAK3, PMS2, TNFRSF13B, TP53	-	AP3B1, BRCA2, CD46, CTC1, DCLRE1C, FANCA, G6PD, GAB2, HAVCR2, IFNA21, IFNA4, IFNA6, IFNB1, JAK3, KMT2D, LY9, PMS2, SIGLEC15, SOCS1, SPINK5, TLR3, TNFRSF13B, TP53
*Type 2 interferon signaling pathway*	HLA-A, IRF3, TP53	–	HLA-A, IRF3, STXBP2, TLR3, TP53
Pathways identified both by genes described on the present study and previously described genes			
*Adaptive immune B cell response*	C6, C8A, CD46, CFHR1, CFHR3, CFHR5, DCLRE1C, HLA-A, IFNA21, JAK3, MASP2, PRF1, TNFRSF13B, TP53	CD84, DOCK8, IFNA21, IFNA4, IFNA6, IFNB1, LY9, UNC13D	AP3B1, C6, C8A, CD46, CD84, DCLRE1C, DOCK8, ERAP1, HLA-A, IFNA21, IFNA4, IFNA6, IFNB1, JAK3, LY9, MASP2, PRF1, RAB27A, SOCS1, TNFRSF13B, TP53, UNC13D
*Adaptive immune T cell response*	C6, C8A, CD46, CFHR1, CFHR3, CFHR5, FANCA, HAVCR2, HLA-A, IFNA21, JAK3, MASP2, PRF1, SPINK5, TNFRSF13B	CD84, DOCK8, IFNA21, IFNA4, IFNA6, IFNB1, LY9, LYST, RAB27A, UNC13D	AP3B1, C6, C8A, CD46, CD84, DOCK8, ERAP1, FANCA, HAVCR2, HLA-A, IFNA21, IFNA4, IFNA6, IFNB1, JAK3, LY9, LYST, MASP2, PRF1, RAB27A, SOCS1, SPINK5, TNFRSF13B, TP53, UNC13D
*Cytokine-mediated signaling pathways*	CD46, CLPB, HAVCR2, HLA-A, IFIH1, IRF3, JAK3	CD84, CYBB, DOCK8, GAB2, IFIH1, IFNA21, IFNA4, IFNA6, IFNAR1, IFNAR2, IFNB1, IL22RA2, IRAK3, IRF3, LY9, NLRP12, NLRP2, SOCS1, STXBP2, TLR3, TLR6, TRAF3	CD46, CD84, CLPB, CYBB, DOCK8, GAB2, HAVCR2, HLA-A, IFIH1, IFNA21, IFNA4, IFNA6, IFNAR1, IFNAR2, IFNB1, IL22RA2, IRAK3, IRF3, JAK3, LY9, NLRP12, NLRP2, SOCS1, STXBP2, TLR3, TLR6, TNFRSF13B, TP53, TRAF3
*DNA-related processes*	BRCA2, CTC1, DCLRE1C, ERCC6L2, FANCA, IRF3, PMS2, POLE, RAD51, TERT, TP53	IRAK3, NLRP12, NLRP2, TLR3, TLR6, TRAF3	BRCA2, CTC1, DCLRE1C, ERCC6L2, FANCA, HAVCR2, IRAK3, IRF3, NLRP12, NLRP2, PMS2, POLE, RAD51, TERC, TERT, TLR3, TLR6, TP53, TRAF3, XIAP
*Response to bacterium*	HAVCR2, SPINK5	IFI44, IFNAR1, IRAK3, IRF3, LYST, SNX3, TLR3, TLR6	ERAP1, HAVCR2, HLA-A, IFI44, IFNAR1, IRAK3, IRF3, LYST, SNX3, SPINK5, TLR3, TLR6
*Response to virus*	CD46, CLPB, FCN3, HAVCR2, HLA-A, IFIH1, IFNA21, IRF3, PRF1, TP53	EXOSC5, IFI44, IFI44L, IFIH1, IFNA4, IFNA6, IFNA21, IFNAR1, IFNAR2, IFNB1, IRAK3, IRF3, LGALS8, LYST, RNASE2, TLR3, TRAF3, UNC13D	CLPB, EXOSC5, FCN3, HAVCR2, HLA-A, IFI44, IFI44L, IFIH1, IFNA21, IFNA4, IFNA6, IFNAR1, IFNAR2, IFNB1, IRAK3, IRF3, LGALS8, LYST, PRF1, RNASE2, STXBP2, TLR3, TP53, TRAF3, UNC13D
*Type 1 interferon signaling pathway*	HAVCR2, HLA-A, IFIH1, IFNA21, IRF3	IFIH1, IFNA21, IFNA4, IFNA6, IFNAR1, IFNAR2, IFNB1, IRF3, TLR3, TRAF3	HAVCR2, HLA-A, IFIH1, IFNA21, IFNA4, IFNA6, IFNAR1, IFNAR2, IFNB1, IRF3, TLR3, TRAF3
Pathways identified exclusively by previously described genes			
*Blood coagulation*	–	AP3B1, DOCK8, IFNA21, IFNA4, IFNA6, IFNB1, PEAR1, RAB27A	AP3B1, DOCK8, IFNA21, IFNA4, IFNA6, IFNB1, PEAR1, RAB27A
*Myeloid leukocyte activation*	-	CD84, CYBB, GAB2, RAB27A, RNASE2, STXBP2, TLR3, TLR6, UNC13D	AP3B1, C6, C8A, CD46, CD84, CYBB, DCLRE1C, DOCK8, FANCA, GAB2, HAVCR2, HLA-A, IFNA21, IFNA4, IFNA6, IFNB1, JAK3, LY9, LYST, MASP2, PRF1, RAB27A, RNASE2, SIGLEC15, SOCS1, SPINK5, STXBP2, TLR3, TLR6, TNFRSF13B, TP53, UNC13D
*NK cell activation*	–	IFNA21, IFNA4, IFNA6, IFNB1, LYST, RAB27A, UNC13D	IFNA21, IFNA4, IFNA6, IFNB1, LYST, RAB27A, UNC13D
*Regulation of inflammatory pathways*	-	AP3B1, CD163, CD84, CYBB, IFIH1, IFNA21, IFNA4, IFNA6, IFNAR1, IFNAR2, IFNB1, IL22RA2, IRAK3, IRF3, NLRP12, NLRP2, SOCS1, TLR3, TLR6, TRAF3, UNC13D, XIAP	AP3B1, CD163, CD84, CYBB, FANCA, HAVCR2, IFIH1, IFNA21, IFNA4, IFNA6, IFNAR1, IFNAR2, IFNB1, IL22RA2, IRAK3, IRF3, JAK3, NLRP12, NLRP2, SOCS1, TLR3, TLR6, TRAF3, UNC13D, XIAP
*Secretion and vesicle dependent transport*	–	AP3B1, CD163, CD84, CYBB, GAB2, GOLGA4, IRF3, LYST, NLRP12, PEAR1, PLIN3, RAB27A, RNASE2, SNX3, SOCS1, STXBP2, UNC13D	CD84, CFTR, GAB2, IRF3, NLRP12, RAB27A, SOCS1, STXBP2, UNC13D
*Type 3 interferon signaling pathway*	-	IFIH1, TLR3	IFIH1, TLR3
Pathways identified exclusively by combining genes described on the present study and previously described genes			
*Angiogenesis*	-	-	C6, CYBB, ERAP1, TERC, TERT, TLR3
*Cell adhesion*	–	–	AP3B1, CD46, DOCK8, HAVCR2, HLA-A, IFNB1, JAK3, SOCS1, SPINK5, UNC13D, ZFHX3
*Exocytosis*	-	-	CD84, CFTR, GAB2, RAB27A, STXBP2, UNC13D

Notably, the 38 genes with CV described in our study are involved in seven major pathways that previously identified genes are also involved in, namely (1) Adaptive immune T cell response, (2) Adaptive immune B cell response, (3) DNA related processes, (4) Response to virus, (5) Response to bacteria, (6) Cytokine-mediated signaling pathways, and (7) Type 1 interferon-signaling pathway. Additionally, three pathways – (1) Complement system, (2) Hematopoiesis and Immune system development, and (3) Type 2 interferon signaling pathway – were first identified in the present study. Our data suggest that in addition to these previously identified pathways, complement system, hematopoiesis and immune system development, and Type 2 interferon signaling pathway impairment may also play a role in MIS-C pathogenesis.

Following this analysis, we combined the genes with CV identified in our analysis with previously described genes and ran a following unsupervised enrichment analysis of BP to search for possible still unidentified pathways. When taken together, the 78 genes clustered into 180 Biological Processes with statistical significance, from which 44 were not detected when considering only the set of genes we identified or the set of previously described genes. Upon supervised analysis of relevant pathways, six of these BPs could be further clustered into three major pathways, namely (1) Angiogenesis, (2) Cell adhesion, and (3) Exocytosis (**Figure 1D**, **Table 3** and **Supplementary Table 6**).

## 4 Discussion

Although the pathophysiology of MIS-C remains unclear, several hypotheses have emerged, including immune dysregulation, autoantibody involvement, antibody-dependent enhancement (ADE), and superantigen-mediated immune activation (Cheng and Zhang, 2020; Consiglio et al., 2020; Kouo and Chaisawangwong, 2021; Ramaswamy et al., 2021; Morita et al., 2022). Also, there are indications that genetic factors may play a role in the pathogenesis of MIS-C, at least in a fraction of the patients, such as the report of siblings with clinical manifestations of MIS-C occurring almost simultaneously (Lim et al., 2021), the similarities with DK and LHH, in which the participation of genetic factors is already established, and the fact that it is a rare complication of SARS-CoV-2 infection (Fujita et al., 1989; Uehara et al., 2003; Sancho-Shimizu et al., 2021).

The present study identified pathogenic or likely pathogenic variants in 29 out of 30 MIS-C patients (96.6%), with a total of 50 CV across 38 different genes (**Table 2**). Notably, several genes, including *HLA-A, PRF1, G6PD*, had CVs detected in more than one patient. These findings support the hypothesis that genetic variants may be involved in the pathophysiology of MIS-C. To our knowledge, this is the largest MIS-C cohort that underwent genetic evaluation in Latin-American patients. The data reported here are similar to the largest cohort of MIS-C cases reported so far (Belay et al., 2021), with 1733 US patients in terms of number of days with fever, prevalence of signs, symptoms, and laboratory findings, as well as the frequency of serologic test and RT-PCR positivity for SARS-CoV-2. Concerning male-female rate, there is a slight predominance of males in the literature. Interestingly, our cohort has a peculiar female predominance. This difference likely does not reflect selection bias, as all patients identified at participating centers who met the MIS-C definition criteria were included in the study without any secondary selection.

A notable finding in our cohort is that only 59.2% of the cases had a traceable history of known contact with a suspected or confirmed case of COVID-19. It is possible that the signs and symptoms of COVID-19 were not appreciated by the contact persons of these children, or even that the individuals who transmitted the virus to the patients were asymptomatic and therefore did not realize they were infected, or did not inform these families about the diagnosis of the disease.

### 4.1 Gene sequencing and MIS-C

In the search for genetic risk factors in patients with MIS-C, one of the strategies has been WES or the use of gene panels to identify deleterious variants in genes related to immune processes. In this sense, Chou et al. (Chou et al., 2021) were pioneers in describing the presence of rare variants in *XIAP*, *CYBB*, and *SOCS1* genes (Lee et al., 2020) in 3 patients diagnosed with MIS-C. Along with the same line, a homozygous variant in the *IFNAR1* gene was reported in a patient diagnosed with MIS-C who had consanguineous parents (Abolhassani et al., 2022b). The authors also described other variants identified in genes related to IEI, including *MASP1*, *POLE2*, and *KMT2D*. Interestingly, we identified CV in *MASP2*, *POLE1*, and *KMT2D* (a different variant from the one identified in the cited study). In 2022, the same research group published a study where 31 patients previously suspected of heaving IEI (but lacking molecular diagnosis) were evaluated after presenting COVID-19 complications, including MIS-C. The cohort had four patients with MIS-C in addition to the one previously described. Two patients had *CFH* variants. A variant in *TBX1* and one in *UNG* were identified in the two remaining patients (Abolhassani et al., 2022a). Of note, we observed CV in *CFHR1*, *CFHR3*, and *CFHR5*, which as *CFH*, are also part of the Human Factor H Gene Cluster.

A case report (Vagrecha et al., 2021), described a patient bearing HLH due to a homozygous frameshift mutation of the gene encoding syntaxin 11 (*STX11*). The subject achieved remission with anakinra monotherapy but subsequently developed an acute SARS-CoV-2 infection followed by a possible MIS-C, both responsive to increased anakinra dosing. A following study involving MIS-C and HLH-related genes described variants in IEI and LHH-related genes in 74% of the patients (29/45) with MIS-C (Vagrecha et al., 2022). One of the genes in which a rare CV (rs779584225) was identified in that study (*PRF1*) also appears among the genes of interest in our cohort. In addition, they also described variants in another four genes we identified CV in our patients (*DCLRE1C*, *IFIH1*, *TNFRSF13B*, *SH3BP2*) (Vagrecha et al., 2022).

A further study involving 45 Middle Eastern children with MIS-C and 25 controls identified rare and probably deleterious variants in 42% of the patients, in 16 genes related to the immune system. Interestingly, in the same study, two CVs that were also identified in our cohort were described (Abuhammour et al., 2022): one in the *IFIH1* gene (rs35337543) and one in the *IFNA21* gene (rs146777169).

Complement-related genes were investigated in 71 pediatric patients from Greece with either MIS-C (7 patients) or COVID-19 (64 patients). Notably, nine patients harbored the same *FCN3* variant identified in three of our patients (rs532781899), four in the non-admitted COVID-19 group and 5 in the admitted group, but none in the MIS-C group (Gavriilaki and Tsiftsoglou, 2022). Also, five patients harbored the same *MASP2* variant identified in two of our patients (rs72550870). However, once more, none of the patients of the MIS-C group harbored the variant. Despite that, the authors themselves recognized that the size of the MIS-C group was relatively small for conclusions about the absence of these variants. It is also worth noting that 22 patients harbored the rs12614 *CFB* variant identified in P12, including four of the seven MIS-C patients (Gavriilaki and Tsiftsoglou, 2022).

More recently, next-generation sequencing (NGS) was performed in samples from 37 MIS-C children at hospital admission and 24 healthy controls from Italy for a panel of 386 genes related to autoimmune diseases, autoinflammation, and primary immunodeficiencies (Gelzo et al., 2022). Similarly, to the present study, the authors identified variants in 34 genes, and 83.3% of patients had at least one variant. Most genes were related to autoimmune diseases like *ATM*, *NCF1*, *MCM4*, *FCN3*, and *DOCK8* or autoinflammatory diseases associated with the release of IFN-gamma, such as *PRF1*, *NOD2*, and *MEF* (Gelzo et al., 2022). Interestingly, three variants identified by the authors were also present in our cohort: *PRF1* rs35947132, *FCN3* rs532781899, and *IFIH1* rs35337543. Abuhammour et al. also identified the latter, as seen above (Abuhammour et al., 2022). It is worth noting that 2/30 patients in our cohort and 5/30 patients reported by Gelzo et al. (Gelzo et al., 2022) had the same uncommon *PRF1* variant (c.272C>T). Although the pathogenicity of this variant is unclear as it is relatively prevalent (AF of 2,8% on GnomAD aggregated), it leads to reduced stability and abnormal traffic of the protein, leading to impaired NK cell cytotoxic function (Voskoboinik et al., 2007; House et al., 2015; Cabrera-Marante et al., 2020).

In another recent study that enrolled 558 MIS-C patients from different ethnicities, 16 different variants were identified in 12 unrelated children who harbor homozygous or compound heterozygous variants in the genes *OAS1*, *OAS2*, OAS3 or *RNASE*, five of which had rare homozygous variants predicted as loss-of-function. However, none of our patients have any of the variants reported (Lee and Le Pen, 2023).

To our knowledge, apart from the present study, the single study that performed WES in Brazilian children with MISC investigated 16 patients (Santos-Rebouças et al., 2022). They screened for very rare variants (global minor allele frequency less than 0.01) previously associated with any known disease, including those unrelated to MISC or SARS-CoV-2 infection. They identified ten very rare variants in eight genes (*FREM1*, *MPO*, *POLG*, *C6*, *C9*, *ABCA4*, *ABCC6*, and *BSCL2*) as promising candidates that could be related to a higher risk of MISC development. From those, seven are classified as pathogenic, and three variants of uncertain significance (VUS) of interest. Interestingly, two of our patients harbor CVs predicted to be pathogenic in their cohort’s most frequently altered gene, *ABCA4* (3 patients bearing three different CVs). The CV found in our p4 (*ABCA4* rs148460146) is in the nucleotide-binding domain (NBD), as the pathogenic variant found in their EXOC13. The second pathogenic ABCA4 CV found in our cohort (in p25) is in the extracellular domain two (ECD2), whose function is currently not fully understood (*ABCA4* rs61750145) (Xie et al., 2021). Another patient in our cohort also harbors a CV in another gene with CV described by them, *ABCC6*. Although it is not the same variant (our patient harbors the *ABCC6* rs72653706, and theirs harbors the *ABCC6* rs63750759), both are classified as pathogenic and associated with Pseudoxanthoma Elasticum (Bergen et al., 2000). The variants in *ABCA4* are associated with retina, macula, and cone-rod dystrophy, which results in vision loss (Lin et al., 2018). However, these CVs are not among our main findings because neither is associated with MISC, KD, severe COVID-19, or any IEI, and their association with MISC is still unclear. Another similarity between both studies is that, as we, they also detected a CV in the *C6* gene. In their study, one patient harbored the CV *C6* rs375762365; in ours, one patient harbored the CV *C6* rs61469168. Although it is not the same variant, both are frameshift mutations, which usually yield a loss of function effect.

Taken together, these findings reinforce the hypothesis that genes previously related to immune processes and, more specifically, IEI may underlie the clinical manifestations of MIS-C.

### 4.2 Gene sequencing and MIS-A

The investigation of possible genetic factors in the pathophysiology of MIS-A through WES has also been described in the scarce literature currently available. In one study, 12 rare and potentially disease-causing VUS were found in 5 patients diagnosed with MIS-A (Ronit et al., 2021), including an *ERAP1* variant, a gene that also had a CV identified in our cohort.

### 4.3 HLA and MIS-C

Another strategy used in the investigation of children and adolescents with MIS-C was evaluating the association of the disease with specific HLA alleles in 2 cohorts of Italian patients. In those studies, it was observed that the combination of the alleles HLA-A*02, B*35, and C*04 was more frequent in patients with severe MIS-C, reinforcing the existence of genetic susceptibility related to the disease (Porritt et al., 2021; Sacco and Castagnoli, 2022). Interestingly, in our cohort, one female patient (P4) was identified with this combination of alleles, and is of Italian descent (**Supplementary Table 7**). Also, 9/30 patients harbor a CV in HLA-A.

### 4.4 Functional enrichment analysis

**Figure 2:**
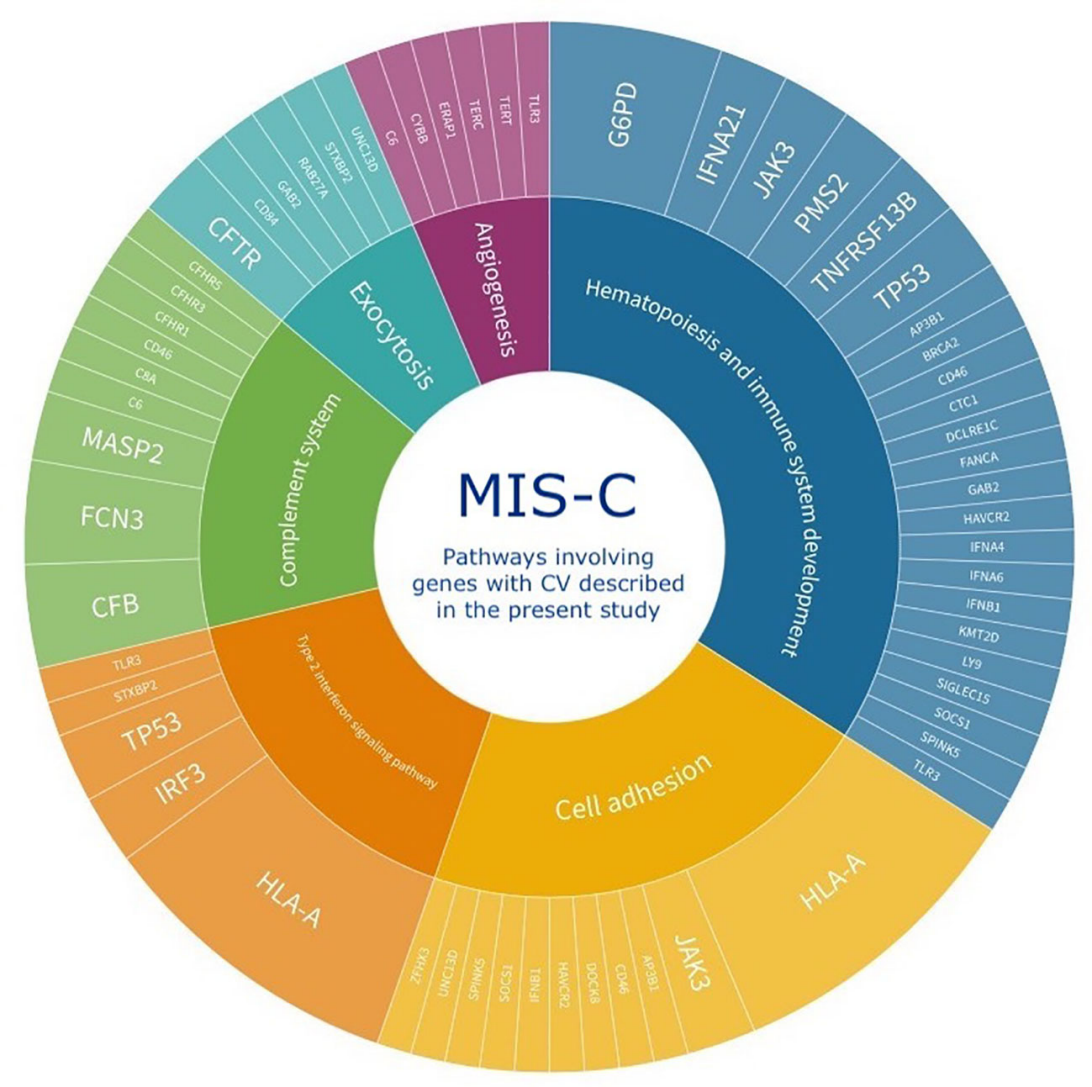
Schematic representation of the six major pathways identified by functional relationships between MISC patients’ genes with CV described by the present study. Three of these are uniquely identified when genes previously reported and genes in our study are analyzed together (Angiogenesis, Cell adhesion, and Exocytosis) and three others emerged exclusively from the analysis of the genes with CV in our patients (Complement system, Hematopoiesis and Immune system development, and Type 2 interferon signaling pathway). The inner circle depicts the pathways and the outer circle the genes with CV involved in each pathway. The size of each pathway in the inner circle is proportional to the number of patients with a respective gene with CV.

Using the gene ontology (GO) enrichment analysis, the functional relationships between genes with CV and the enriched biological processes (BP) were analyzed. With this information combined with supervised annotation of relevant pathways, Circos graphs were generated (**Figure 1B**). Most of the identified pathways were consistent with a potential over-persistent state of inflammation, possibly due to COVID-19 infection, which could lead to the onset of MIS-C. Our study identified 13 potentially relevant pathways in the context of MIS-C, seven in common with the analysis of genes previously reported in the literature and six not previously described by the WES approach (**Figure 2**). Three of these are uniquely identified when genes previously reported and genes in our study are analyzed together, and three others emerged exclusively from the analysis of the genes with CV in our patients (**Supplementary Table 6**). Some of these pathways have also been highlighted in the literature and may shed light on the mechanisms that lead to this disease, as discussed below.

The complement system is one of the most activated intracellular pathways during the COVID-19 antiviral response, and deregulation of this pathway has been described in MIS-C (Ali et al., 2021; Defendi et al., 2021; Syrimi et al., 2021; Afzali and Noris, 2022; Kumar et al., 2022). Interestingly, 14/30 of our patients had a CV in complement system genes.

Type I interferons (IFN) have a crucial role in antiviral response and severe COVID-19 (Bastard and Rosen, 2020; Zhang and Bastard, 2020; Chou et al., 2021; Tovo and Garazzino, 2021; Abolhassani et al., 2022b). Patients with MIS-C or severe COVID-19 harbor reduced expression of type I IFN-stimulated genes (ISGs), whereas children with mild COVID-19 exhibit an increased expression pattern (Tovo and Garazzino, 2021). In line with these findings, studies in adults have linked this reduction to a persistent viral load and consequent hyperinflammatory state (Trouillet-Assant et al., 2020). In our cohort, 18/30 patients harbor a CV in Response to Virus Pathways related genes.

In addition to type I IFN involvement in MIS-C, type II IFN-dependent and NF-κB-dependent signatures, matrisome activation, increased cell adhesion biomarkers, and increased levels of circulating spike protein have also been detected in MIS-C (Lee et al., 2020; Chou et al., 2021; Sacco and Castagnoli, 2022). Notably, one of the studies that detected increased type II IFN-dependent signaling in MIS-C also described increased cell adhesion biomarkers, one of the major pathways we detected in our analysis when combining previously described genes with genes detected in our present study (Sacco and Castagnoli, 2022). A total of 12/30 patients in our cohort harbor CV in genes associated with the type II IFN pathway.

Many hematopoietic findings are frequently observed in MIS-C patients, such as leukocytosis, lymphopenia, and thrombocytosis/thrombocytopenia (Belay et al., 2021). Also, very small embryonic-like stem cells (VSEL) and hematopoietic stem cells (HSC) express ACE2, SARS-CoV-2 entry receptor. Hence, VSELs residing in developed tissues could be damaged by SARS-CoV-2, with remote effects on tissue/organ regeneration, which is a feature of MIS-C (Ratajczak et al., 2021). Therefore, variants in genes that disrupt hematopoiesis and immune system development might impair response to SARS-CoV-2, favoring the development of MIS-C. In our study, 14/30 patients harbor CV in hematopoiesis and immune system development related genes.

Finally, our results also support a role in MIS-C of variants in genes involved in multiple pathways or haplotypes. For example, all 5/30 patients with *PFR1* variants detected in our study also harbored variants in at least one complement system gene, and co-occurrence of CV in *PFR1* with CV in other genes was also observed in the other studies that reported CV in *PRF1* (Gelzo et al., 2022; Vagrecha et al., 2022).

### 4.5 Involvement of genetic factors in MIS-C

The clinical phenotype of MIS-C is variable, and several categorizations of these patients into subgroups have been proposed (Godfred-Cato et al., 2020; Whittaker et al., 2020; Harwood et al., 2021). Also, because of this variability, it is speculated that at least a portion of patients with MIS-C may have an underlying IEI, either of monogenic, digenic, or even oligogenic origin (Sancho-Shimizu et al., 2021). Thus, genetic studies such as whole genome sequencing or WES are important to better understand this disease pathophysiology. Furthermore, to further tackle which are the determinant factors in the development of MIS-C, other strategies should be employed, such as an *in-depth* genetic investigation through HLA analysis, evaluation of the expression of genes of interest using proteomics, and search for pathways that may be hyper- or hypo-activated, with the use of transcriptomics using bulk and single-cell analysis.

The limitations of the present study include the absence of a control group, the non-consideration of VUS in the analysis, and the absence of functional tests, which are necessary to determine the actual damage caused by the variants described. By searching for variants previously identified as pathogenic or likely pathogenic, we partially addressed this limitation, but further studies are needed to better understand our findings so that they can be translated into clinical advice. It is also important to note that several of the CVs described were identified in only one subject, and more than one CV was found in most subjects. Based on the limitations mentioned above, it is currently not possible to conclude that the cited variants are directly involved in the pathophysiology of MIS-C. As a rare disease, a worldwide multicentric effort would be ideal for strengthening the findings from this cohort and other studies.

What appears to be shared by all WES and other genetic studies carried out on MIS-C so far is that children with MIS-C may take benefit from an approach such as the search for disease-causing mutations based on an IEI-associated gene panel. In addition, early detection before the onset of the disease may help prevent severe clinical scenarios for these children and enable better clinical management.

## 5 Conclusions

In our cohort, 29/30 MIS-C patients harbored uncommon and rare variants in genes related to immune system disorders. Our results support the hypothesis that MIS-C has an immune-genetic basis, as summarized in **Figure 2**. Importantly, however, other unidentified genes or factors are also likely to be involved.

To our knowledge, this is the largest MIS-C cohort that underwent genetic evaluation in Latin American patients. In a second vein, our study strengthens the need for larger genomic studies in children with MIS-C to identify other possible variants involved in the pathogenesis of the disease, to better understand the role of genetics in MIS-C, and eventually to provide assistance in the clinical management of patients in a higher risk of poor outcomes.

Many IEIs are silent during the first years of life, and several phenotypes appear after exposure to particular infectious agents. Our data support the use of WES as a diagnostic tool for children with this rare COVID-19 complication. This strategy may help to pinpoint the genetic impairments contributing to the development of the MIS-C phenotype in children, identify children at a higher risk of worse outcomes, and provide guidance to narrow treatment decisions.

## Data availability statement

The data presented in the study are deposited in the ARCA Dados Fiocruz repository, accession doi: https://doi.org/10.35078/RDZHNL.

## Ethics statement

The studies involving human participants were reviewed and approved by Internal Review Board (IRB) of Instituto D’Or de Pesquisa e Ensino - IDOR (Proponent Institution) under CAAE n° 30272920.0.1001.5249. Written informed consent to participate in this study was provided by the participants’ legal guardian/next of kin. Written informed consent was obtained from the individual(s), and minor(s)’ legal guardian/next of kin, for the publication of any potentially identifiable images or data included in this article.

## Author contributions

Conceptualization, BR, AP-B, and ZV. Methodology, BR, and ZV. Formal analysis, BR, RSF, FF, HF, DP, ISF, DLMF, and OC-M. Investigation, BR, FC, MZ, DM, ACB, WS, JR, FO, ANB, RZ, MS, MO, and GR-S. Resources, FF, DP, MZ, OC-M, MS. Data curation, BR, FF, and RSF. Writing—original draft preparation, BR, and RSF. Writing—review and editing, FC, DM, ACB, WS, AP-B, and ZV. Visualization, BR, RSF, DP, ISF, and DLMF. Supervision, AP-B and ZV. Project administration, ZV. Funding acquisition, FP, OC-M, AP-B, and ZV. All authors contributed to the article and approved the submitted version.
