# Supplementary material for: Rare genetic variants involved in multisystem inflammatory syndrome in children: a multicenter Brazilian cohort study
Source: Front Cell Infect Microbiol. 2023 Jul 31;13:1182257. doi: 10.3389/fcimb.2023.1182257 (PMC10426286; doi:10.3389/fcimb.2023.1182257)
Supplement: Supplementary file 1 [file Table_1.docx]

Supplementary Material

Rare Genetic Variants Involved in Multisystem Inflammatory Syndrome in Children (MIS-C): a multicenter Brazilian cohort study

**Bárbara C. S. Reis^†1,2,3^, Roberta Soares Faccion^†1,2^, Flavia Amendola Anisio de Carvalho^1,3^, Daniella Campelo Batalha Cox Moore^4,5^, Maria Celia Chaves Zuma^1,2^, Desirée Rodrigues Plaça ^6,7^, Igor Salerno Filgueiras^8^, Dennyson Leandro M. Fonseca^9^, Otavio Cabral-Marques^6,8,9,10,11,12^, Adriana Cesar Bonomo^13,14,15,16^, Wilson Savino^13,14,15,16^, Flávia Cristina de Paula Freitas^17^, Helisson Faoro^17^, Fabio Passetti^17^, Jaqueline Rodrigues Robaina^18^, Felipe Rezende Caino de Oliveira^19^, Ana Paula Novaes Bellinat^20^, Raquel de Seixas Zeitel^21^, Margarida dos Santos Salú^1,2,18^, Mariana Barros Genuíno de Oliveira^18^, Gustavo Rodrigues-Santos^18^, Arnaldo Prata-Barbosa^18,22^, Zilton Vasconcelos^2*^**

**Correspondence:** Zilton Vasconcelos: zilton.vasconcelos@fiocruz.br

| ***Supplementary Table 1: Patients’ data collected through the RedCap form.*** | *Page 2* |
| --- | --- |
| ***Supplementary Table 2: Genes included in each panel used in the analysis.*** | *Page 6* |
| ***Supplementary Table 3: Websites of allele frequency databases and predictors tools consulted in the present study.*** | *Page 7* |
| ***Supplementary Table 4: Predominant VOC circulating in Brazil at the onset of each MISC case analyzed.*** | *Page 8* |
| ***Supplementary Table 5: Genes with CV and patients clinical phenotype*** | *Page 9* |
| ***Supplementary Table 6: String Enrichment non-supervised analysis of Biological Processes involving the identified genes, followed by supervised further categorization.*** | *Page 11* |
| ***Supplementary Table 7: Frequency of HLA-A, B and C in MIS-C patients.*** | *Page 12* |
|  |  |

Supplementary Table 1: Patients’ data collected through the RedCap form.

| Group | Variables | Fields |
| --- | --- | --- |
| Demographic |  |  |
|  | Record ID | Numbers |
|  | Date of Filing | Numbers |
|  | Hospital Name | Text |
|  | Patient Initials | Text |
|  | Patient Hospital ID | Numbers |
|  | Patient Date of Birth | Numbers |
|  | Age (years, months and days) | Numbers |
|  | Race | White/Black/Asian/Indigenous/Brown |
|  | Sex | Male/Female |
|  | Weight | Numbers |
|  | Height | Numbers |
|  | ICU Admitted? | Y/N |
|  | Comorbidities | Prematurity, asthma, obesity, onco-hematological disease, kidney disease, liver disease, rheumatologic disease, cardiac disease, malnutrition, neurologic disease, chronic lung disease, other |
|  | Describe comorbidities | Text |
| Clinical Data |  |  |
|  | Date of Hospital Admission | Numbers |
|  | Date ICU Admission | Numbers |
|  | Contact with COVID-19? | Y/N |
|  | Who is the Suspected/Confirmed Case? | Text |
|  | MIS-C Phenotype | Incomplete Kawasaki disease, toxic shock syndrome, acute cardiac dysfunction, Kawasaki-like disease, macrophage activation syndrome |
|  | Clinical Signs on Admission | Fever, cough, tachypnea, respiratory distress, SO2 < 92%, cyanosis, grunting, food refusal, dehydration, diarrhea, emesis, other |
|  | Other Signs | Text |
|  | Plain Radiography Results on Admission | Text |
|  | Total Fever Days | Numbers |
|  | Additional Symptoms | GIT symptoms, respiratory symptoms, skin rash, conjunctivitis, mucositis, lymphadenopathy, irritability |
|  | GIT Symptoms | Abdominal pain, diarrhea, emesis, enteritis, lymphadenopathy (suggestive image), other (describe) |
|  | Respiratory Symptoms | Cough, thoracic pain, tachypnea, dyspnea |
|  | Shock Signs? | Y/N |
|  | Describe Shock Signs | Hypotension (age reference), tachycardia (age reference), slow capillary refill time, pallor, cold extremities, urinary output < 2 mg/kg/h, metabolic acidosis, high lactate, acute kidney injury, acute hepatic injury, supplementary oxygen need |
|  | Lower Systolic Blood Pressure | Numbers |
|  | Lower Diastolic Blood Pressure | Numbers |
|  | Higher Cardiac Rate | Numbers |
|  | Treatment | Immunoglobulin, glucocorticoid therapy, anticoagulant therapy, acetylsalicylic acid, other |
|  | Total immunoglobulin Dose | Numbers |
|  | Glucocorticoid Used | Text |
|  | Glucocorticoid Dose | Numbers |
|  | Describe Other Medications | Text |
|  | Patient Received Blood Transfusion? | Y/N |
|  | Blood Product Received | Text |
|  | Date of Transfusion | Numbers |
|  | Total Supplementary Oxygen (Days) | Numbers |
|  | Needed Invasive Mechanical Ventilation? | Y/N |
|  | Total Invasive Mechanical Ventilation (Days) | Numbers |
| Laboratory Data |  |  |
|  | Leucocytes on Admission | Numbers |
|  | Lower Leucocyte Count | Numbers |
|  | Higher Leucocyte Count | Numbers |
|  | Lymphopenia? | Y/N |
|  | Lower Lymphocytes Count | Numbers |
|  | Platelets Count on Admission | Numbers |
|  | Lower Platelets Count | Numbers |
|  | Higher Platelets Count | Numbers |
|  | Reactive C Protein on Admission | Numbers |
|  | Higher Urea | Numbers |
|  | Higher Creatinine | Numbers |
|  | Higher Lactic Dehydrogenase | Numbers |
|  | Higher ALT | Numbers |
|  | Higher AST | Numbers |
|  | Higher GGT | Numbers |
|  | Lower Albumin | Numbers |
|  | Higher Total CK | Numbers |
|  | CK-MB on Admission | Numbers |
|  | Pro-BNP on Admission | Numbers |
|  | Troponin on Admission | Numbers |
|  | Ferritin on Admission | Numbers |
|  | Elevated Inflammatory Markers? | Y/N |
|  | ESR | Numbers |
|  | PCR | Numbers |
|  | Pro-calcitonin | Numbers |
|  | Ferritin | Numbers |
|  | Elevated Cardiac Dysfunction Markers? | Y/N |
|  | CK-MB | Numbers |
|  | Pro-BNP | Numbers |
|  | Troponin | Numbers |
|  | ECG Alterations? | Y/N |
|  | Describe ECG | Text |
|  | Coagulopathy? | Y/N |
|  | APTT | Numbers |
|  | INR | Numbers |
|  | PT | Numbers |
|  | D-dimer | Numbers |
|  | Fibrinogen | Numbers |
|  | Echocardiogram Alterations? | Y/N |
|  | Describe Echocardiogram | Text |
|  | Anemia? | Y/N |
|  | Lower Hemoglobin | Numbers |
|  | Lower Hematocrit | Numbers |
|  | Chest CT? | Y/N |
|  | CT Alterations | Text |
|  | COVID-19 Tests on Hospital | Serology (ELISA), rapid serologic test, RT-PCR (nose and throat), RT-PCR (tracheal secretions), RT-PCR (nasopharyngeal secretions) |
|  | Date of Serology (ELISA) | Numbers |
|  | IgA | Y/N |
|  | IgA Values | Numbers |
|  | IgM | Y/N |
|  | IgM Values | Numbers |
|  | IgG | Y/N |
|  | IgG Values | Numbers |
|  | Date of Rapid Serologic Test | Numbers |
|  | IgM | Numbers |
|  | IgG | Numbers |
|  | Date of RT-PCR | Numbers |
|  | RT-PCR Result | Detected/Not detected |
|  | Other COVID-19 Tests | Text |
|  | Date of Blood Sample for Exome/ELISA | Numbers |
|  | IgA Values | Numbers |
|  | IgG Values | Numbers |

Y/N: Yes or No.

**Supplementary Table 2: Genes included in each panel used in the analysis.**

| Inborn Errors of Immunity (IEI) | ACD, **ACP5**, ACTB, **ADA**, **ADA2**, ADAM17, **ADAR**, AICDA, AIRE, AK2, ALPI, AP1S3, **AP3B1**, AP3D1, APOL1, ARHGEF1, ARPC1B, ATG4A, **ATM**, ATP6AP1, B2M, BACH2, BCL10, BCL11B, BLM, BLNK, BRCA1, BRCA2, BRIP1, BTK, C1QA, C1QB, C1QC, C1R, C1S, **C2**, C3, C5, **C6**, C7, **C8A**, C8B, C8G, C9, CARD11, **CARD14**, CARD9, CARMIL2, CASP10, CASP8, CCBE1, CD19, CD247, CD27, CD3D, CD3E, CD3G, **CD40**, CD40LG, CD46, CD55, CD59, CD70, CD79A, CD79B, CD81, CD8A, CDC42, CDCA7, CEBPE, CFB, CFD, **CFH**, CFHR1, CFHR2, CFHR3, CFHR4, CFHR5, **CFI**, CFP, CFTR, CHD7, CIB1, CIITA, CLCN7, **CLPB**, COPA, CORO1A, **CR2**, CSF2RA, CSF2RB, CSF3R, CTC1, CTLA4, CTNNBL1, CTPS1, CTSC, CXCR4, CYBA, **CYBB**, CYBC1, DBR1, **DCLRE1C**, DEF6, DKC1, **DNAJC21**, DNASE1L3, DNASE2, DNMT3B, DOCK2, **DOCK8**, EFL1, ELANE, EPG5, ERBIN, ERCC4, ERCC6L2, EXTL3, FAAP24, FADD, FANCA, FANCB, FANCC, FANCD2, FANCE, FANCF, FANCG, FANCI, FANCL, FANCM, FAS, FASLG, FAT4, FCGR3A, FCHO1, FCN3, FERMT1, FERMT3, FNIP1, FOXN1, FOXP3, FPR1, G6PC3, G6PD, GATA2, GFI1, GINS1, HAVCR2, HAX1, HELLS, HYOU1, ICOS, ICOSLG, **IFIH1**, **IFNAR1**, **IFNAR2**, IFNG, IFNGR1, IFNGR2, IGHM, IGKC, IGLL1, IKBKB, **IKBKG**, IKZF1, IL10, **IL10RA**, IL10RB, IL12B, IL12RB1, IL12RB2, IL17F, IL17RA, IL17RC, IL18BP, **IL1RN**, IL21, IL21R, IL23R, IL2RA, IL2RB, IL2RG, IL36RN, IL6R, IL6ST, IL7R, INO80, IRAK1, IRAK4, IRF2BP2, IRF3, IRF4, **IRF7**, IRF8, **IRF9**, ISG15, ITCH, ITGB2, ITK, JAGN1, JAK1, JAK3, KDM6A, KMT2A, **KMT2D**, KRT20, LAMTOR2, LAT, LCK, LCP2, LIG1, **LIG4**, **LPIN2**, **LRBA**, LSM11, **LYST**, MAD2L2, MAGT1, **MALT1**, MAP1LC3B2, MAP3K14, MAPK8, MASP2, MCM10, **MCM4**, **MEFV**, MOGS, MRTFA, MSH6, MSN, MTHFD1, MVK, MYD88, MYSM1, NBAS, NBN, **NCF1**, **NCF2**, NCF4, NCKAP1L, NCSTN, NFAT5, NFE2L2, NFKB1, NFKB2, NFKBIA, NHEJ1, NHP2, **NLRC4**, **NLRP1**, **NLRP12**, NLRP3, **NOD2**, NOP10, NOS2, NSMCE3, **OAS1**, **ORAI1**, OSTM1, OTULIN, PALB2, PARN, PAX1, **PEPD**, PGM3, **PIK3CD**, PIK3CG, **PIK3R1**, **PLCG2**, PLEKHM1, PMS2, PNP, POLA1, POLD1, POLD2, POLE, POLE2, POLR3A, POLR3C, POLR3F, **PRF1**, PRKCD, **PRKDC**, PSENEN, PSMB8, PSMG2, **PSTPIP1**, PTEN, PTPRC, **RAB27A**, RAC2, RAD51, RAD51C, **RAG1**, RAG2, RANBP2, RASGRP1, RBCK1, REL, RELA, RELB, RFWD3, RFX5, RFXANK, RFXAP, RHOH, RIPK1, **RMRP**, RNASEH2A, RNASEH2B, RNASEH2C, RNF168, RNF31, RNU4ATAC, RNU7-1, RORC, RPSA, RTEL1, SAMD9, SAMD9L, SAMHD1, SBDS, SEC61A1, **SEMA3E**, SERPING1, SH2D1A, **SH3BP2**, SH3KBP1, SKIV2L, **SLC29A3**, SLC35C1, **SLC37A4**, SLC39A7, SLC46A1, SLC7A7, SLX4, SMARCAL1, SMARCD2, SNORA31, SNX10, **SOCS1**, SP110, SPINK5, SPPL2A, SRP54, SRP72, **STAT1**, **STAT2**, STAT3, STAT5B, STIM1, STK4, STN1, STX11, **STXBP2**, TAP1, TAP2, TAPBP, TAZ, **TBK1**, **TBX1**, TBX21, TCF3, TCIRG1, TCN2, TERC, TERT, TET2, **TFRC**, TGFB1, TGFBR1, TGFBR2, THBD, **TICAM1**, TINF2, TIRAP, **TLR3**, TMC6, TMC8, **TMEM173**, TNFAIP3, **TNFRSF11A**, **TNFRSF13B**, TNFRSF13C, TNFRSF1A, TNFRSF4, TNFRSF9, TNFSF11, TNFSF12, TNFSF13, TOP2B, TP53, **TPP2**, TRAC, **TRAF3**, TRAF3IP2, TREX1, TRIM22, TRNT1, TTC37, **TTC7A**, TYK2, UBA1, UBE2T, UNC13D, **UNC93B1**, **UNG**, USB1, USP18, VPS13B, VPS45, WAS, WDR1, WIPF1, WRAP53, **XIAP**, XRCC2, **ZAP70**, ZBTB24, ZNF341 |
| --- | --- |
| Associated with Kawasaki Disease | ABCC4, ACE, BLK, CAMK2D, CASP3, CCL17, **CD40**, COPB2, CSMD1, **ERAP1**, FCGR2A, FGB, FGF23, HLA-A, IGH, IGHV3OR16-7, IL1A, IL4, IL6, ITPKC, ITPR3, KCNN2, LNX1, LTA, MBL2, MIA-RAB4B, MMP12, MMP3, MPO, NAALADL2, ORAI1, PELI1, SLC11A1, SLC8A1, SMAD3, SMAD5, SNCA, STIM1, TCP1, TGFB2, TGFBR2, **TLR6**, TNF, VEGFA, ZFHX3 |
| Severe COVID-19 | **IFNAR1**, **IFNAR2**, **IKBKG**, **IRF3**, **IRF7**, **IRF9**, **STAT1**, **STAT2**, **TBK1**, **TICAM1**, **TLR3**, TLR7, **TRAF3**, **UNC93B1** |
| Multisystem Inflammatory Syndrome in Children (MIS-C) | **ACP5**, **ADA**, **ADA2**, **ADAR**, **AP3B1**, **ATM**, **CARD14**, CD163, **C2**, **C6**, **C8A**, CD84, **CFH**, **CFI**, **CLPB**, **CR2**, **CYBB**, **DCLRE1C**, **DNAJC21**, DNASE1, **DOCK8**, IFI44, IFI44L, **IFIH1**, IFNA21, IFNA4, IFNA6, IFNAR1, IFNB1, **IKBKG**, **IL10RA**, **IL1RN**, IL22RA2, IRAK3, **KMT2D**, **LIG4**, **LPIN2**, **LRBA**, LY9, **LYST**, **MALT1**, **MCM4**, **MEFV**, **NCF1**, **NCF2**, **NLRC4**, **NLRP1**, **NLRP12**, NLRP2, **NOD2**, **OAS1**, OAS2, OAS3, **ORAI1**, **PEPD**, **PIK3CD**, **PIK3R1**, **PLCG2**, **PRF1**, **PRKDC, PSTPIP1**, **RAB27A**, **RAG1**, **RMRP**, RNASEL, SEMA3E, **SH3BP2**, **SLC29A3**, **SLC37A4, SOCS1**, **STAT1**, **STXBP2**, **TBX1**, TFRC, **TLR6**, **TMEM173**, **TNFRSF13B**, **TNFRSF1A**, **TPP2**, **TTC7A, UNC13D**, **UNG**, **XIAP**, **ZAP70** |
| Multisystem Inflammatory Syndrome in Adults (MIS-A) | **ERAP1**, EXOSC5, FMNL1, GAB2, GOLGA4, LrGALS8, PEAR1, PLIN3, RNASE2, SIGLEC15, SNX3, TECPR1 |

Genes in bold are present in more than one gene panel.

**Supplementary Table 3: Websites of allele frequency databases and predictors tools consulted in the present study.**

| **Allele Frequency Databases** | |
| --- | --- |
| 1000 Genomes Project databse | https://www.internationalgenome.org |
| ABraOM | http://abraom.ib.usp.br/ |
| ExAC/gnomAD | https://gnomad.broadinstitute.org |
| **Predictors** | |
| BayesDel_addAF | https://fengbj-laboratory.org/BayesDel/BayesDel.html |
| CADD | https://cadd.gs.washington.edu/ |
| DANN | https://maayanlab.cloud/datasets2tools/landing/tool/DANN |
| DEOGEN2 | https://openebench.bsc.es/tool/deogen2 |
| EIGEN | http://www.columbia.edu/~ii2135/eigen.html |
| FathmmMKL | https://fathmm.biocompute.org.uk/fathmmMKL.htm |
| LIST-S2 | https://list-s2.msl.ubc.ca/ |
| M-CAP | http://bejerano.stanford.edu/mcap/ |
| MutationAssessor | http://mutationassessor.org/r3/ |
| MutationTaster | https://www.mutationtaster.org |
| Polyphen-2 | http://genetics.bwh.harvard.edu/pph2/ |
| PrimateAI | https://www.nature.com/articles/s41588-018-0167-z |
| SIFT | https://sift.bii.a-star.edu.sg |

**Supplementary Table 4: Predominant VOC circulating in Brazil at the onset of each MISC case analyzed.**

| **Patient** | **Date of MISC onset** | **Predominant VOC in Brazil at that time** |
| --- | --- | --- |
| P1 | 06/12/20 | Wuhan |
| P2 | 10/02/21 | P.1 (Gamma) |
| P3 | 21/06/21 | P.1 (Gamma) |
| P4 | 29/08/20 | Wuhan |
| P5 | 10/01/21 | Wuhan |
| P6 | 07/05/20 | Wuhan |
| P7 | 24/11/20 | Wuhan |
| P8 | 04/06/20 | Wuhan |
| P9 | 08/01/21 | Wuhan |
| P10 | 13/01/21 | Wuhan |
| P11 | 01/05/21 | P.1 (Gamma) |
| P12 | 23/07/20 | Wuhan |
| P13 | 13/01/21 | Wuhan |
| P14 | 20/08/21 | AY (Delta) |
| P15 | 29/01/21 | Wuhan |
| P16 | 02/07/20 | Wuhan |
| P17 | 24/05/20 | Wuhan |
| P18 | 28/07/20 | Wuhan |
| P19 | 01/10/20 | Wuhan |
| P20 | 11/06/20 | Wuhan |
| P21 | 26/04/21 | P.1 (Gamma) |
| P22 | 26/04/21 | P.1 (Gamma) |
| P23 | 25/02/21 | P.1 (Gamma) |
| P24 | 19/06/21 | P.1 (Gamma) |
| P25 | 05/05/21 | P.1 (Gamma) |
| P26 | 22/09/20 | Wuhan |
| P27 | 28/05/21 | P.1 (Gamma) |
| P28 | 14/04/21 | P.1 (Gamma) |
| P29 | 06/05/21 | P.1 (Gamma) |
| P30 | 15/01/22 | BA.1 (Omicron) |

**Supplementary Table 5: *Genes with CV and patients clinical phenotype***

| **Patient** | **Phenotype** | **Gene with CV** | **Gene Panel** |
| --- | --- | --- | --- |
| P1 | Acute Cardiac Dysfunction | CLPB HLA-A JAK3 | IEI Kawasaki IEI |
| P2 | Acute Cardiac Dysfunction | SPINK5 HLA-A | IEI Kawasaki |
| P3 | Incomplete Kawasaki | BRCA2 | IEI |
| P4 | Acute Cardiac Dysfunction | MASP2 IFIH1 | IEI IEI |
| P5 | Acute Cardiac Dysfunction | CFB CTC1 G6PD | IEI IEI  Kawasaki |
| P6 | Kawasaki-like | FCN3 | IEI |
| P7 | Acute Cardiac Dysfunction | TERC APOL1 | IEI IEI |
| P8 | Kawasaki-like | HLA-A | Kawasaki |
| P9 | Incomplete Kawasaki | FANCA HLA-A | IEI Kawasaki |
| P10 | Toxic Shock Syndrome | IRF3 | IEI/COVID-19 |
| P11 | Acute Cardiac Dysfunction | CFB PRF1 PMS2 POLE | IEI IEI  IEI IEI |
| P12 | Acute Cardiac Dysfunction | CFTR PRF1 CFB | IEI  IEI  IEI |
| P13 | Kawasaki-like | C6 CFTR PRF1 | IEI  IEI  IEI |
| P14 | Not Available | HLA-A | Kawasaki |
| P15 | Acute Cardiac Dysfunction | TNFRSF13B | IEI |
| P16 | Kawasaki-like | APOL1 FCN3 | IEI IEI |
| P17 | Kawasaki-like | MASP2 JAK3 | IEI IEI |
| P18 | Kawasaki-like | FCN3 | IEI |
| P19 | Acute Cardiac Dysfunction | CD46 ERCC6L2 HLA-A PRF1 | IEI IEI Kawasaki IEI |
| P20 | Kawasaki-like | TERT PRF1 APOL1 HLA-A ZFHX3 CFHR5 | IEI  IEI IEI Kawasaki Kawasaki IEI |
| P21 | Acute Cardiac Dysfunction | CFHR3 G6PD RAD51 TP53 | IEI  Kawasaki  IEI  IEI |
| P22 | Incomplete Kawasaki | KMT2D TP53 | IEI  IEI |
| P23 | Acute Cardiac Dysfunction | G6PD | Kawasaki |
| P24 | Kawasaki-like | C8A ERAP1 DCLRE1C | IEI  Kawasaki/MIS-C  IEI |
| P25 | Acute Cardiac Dysfunction | HLA-A | Kawasaki |
| P26 | Acute Cardiac Dysfunction | G6PD CFHR1 SH3BP2 HLA-A | Kawasaki  IEI  IEI/MISC-C  Kawasaki |
| P27 | Kawasaki-like | PMS2 RANBP2 | IEI  IEI |
| P28 | Acute Cardiac Dysfunction | G6PD HAVCR2 | Kawasaki  Kawasaki |
| P29 | Incomplete Kawasaki | TNFRSF13B IFNA21 | IEI  MIS-C |
| P30 | Kawasaki-like | - | - |

**Supplementary Table 6: String Enrichment non-supervised analysis of Biological Processes involving the identified genes, followed by supervised further categorization.**

| Pathways | Number of non-supervised Biological Processes involving genes | | |
| --- | --- | --- | --- |
|  | Identified in the present study | Previously described by other groups | Identified in the present study and previously described |
| Our Data exclusive | 8 | - | 1 |
| *Complement system* | 4 | - | 0 |
| *Hematopoiesis and immune system development* | 3 | - | 1 |
| *Type 2 interferon signaling pathway* | 1 | - | 0 |
| Our Data and Literature | 27 | 29 | 12 |
| *Adaptive immune T cell response* | 7 | 2 | 8 |
| *Adaptive immune B cell response* | 2 | 2 | 0 |
| *DNA related processes* | 6 | 1 | 1 |
| *Response to virus* | 7 | 10 | 0 |
| *Response to bacterium* | 1 | 1 | 0 |
| *Cytokine-mediated signaling pathways* | 1 | 8 | 2 |
| *Type 1 interferon signaling pathway* | 3 | 5 | 1 |
| Literature exclusive | - | 19 | 4 |
| *Blood coagulation* | - | 1 | 0 |
| *Myeloid leukocyte activation* | - | 2 | 0 |
| *NK cell activation* | - | 3 | 0 |
| *Secretion and vesicle dependent transport* | - | 3 | 0 |
| *Regulation of inflammatory pathways* | - | 9 | 4 |
| *Type 3 interferon signaling pathway* | - | 1 | 0 |
| Our Data plus Literature | - | - | 6 |
| *Angiogenesis* | - | - | 1 |
| *Cell adhesion* | - | - | 3 |
| *Exocytosis* | - | - | 2 |

**Supplementary Table 7: Frequency of HLA-A, B and C in MIS-C patients.**

| **HLA** | **Frequency** | **Percentage** |
| --- | --- | --- |
| *A*02* | 14 | 46.7 |
| A*30 | 8 | 26.7 |
| A*01 | 8 | 26.7 |
| A*03 | 7 | 23.3 |
| A*68 | 6 | 20.0 |
| A*33 | 5 | 16.7 |
| A*23 | 3 | 10.0 |
| A*11 | 3 | 10.0 |
| A*74 | 2 | 6.7 |
| A*31 | 2 | 6.7 |
| A*36 | 1 | 3.3 |
| A*26 | 1 | 3.3 |
| B*07 | 6 | 21.4 |
| B*15 | 6 | 21.4 |
| B*51 | 5 | 17.9 |
| B*35 | 5 | 17.9 |
| B*14 | 4 | 14.3 |
| B*53 | 4 | 14.3 |
| B*44 | 4 | .14.3 |
| B*57 | 3 | 10.7 |
| *B*42* | 3 | 10.7 |
| B*58 | 2 | 71 |
| B*40 | 2 | 7.1 |
| B*39 | 2 | 7.1 |
| B*49 | 2 | 7.1 |
| B*81 | 1 | 3.6 |
| B*13 | 1 | 3.6 |
| B*08 | 1 | 3.6 |
| B*50 | 1 | 3.6 |
| B*45 | 1 | 3.6 |
| B*18 | 1 | 3.6 |
| B*52 | 1 | 3.6 |
| B*41 | 1 | 3.6 |
| C*07 | 13 | 46.4 |
| C*04 | 9 | 32.1 |
| C*03 | 5 | 17.9 |
| C*08 | 4 | 14.3 |
| C*15 | 4 | 14.3 |
| C*17 | 4 | 14.3 |
| C*16 | 3 | 10.7 |
| C*18 | 3 | 10.7 |
| C*02 | 3 | 10.7 |
| C*05 | 2 | 7.1 |
| C*06 | 2 | 7.1 |
| C*12 | 2 | 7.1 |
| C*01 | 1 | 3.6 |
| C*14 | 1 | 3.6 |
